# 3D confocal laser-scanning microscopy for large-area imaging of the corneal subbasal nerve plexus

**DOI:** 10.1038/s41598-018-25915-6

**Published:** 2018-05-10

**Authors:** Stephan Allgeier, Andreas Bartschat, Sebastian Bohn, Sabine Peschel, Klaus-Martin Reichert, Karsten Sperlich, Marcus Walckling, Veit Hagenmeyer, Ralf Mikut, Oliver Stachs, Bernd Köhler

**Affiliations:** 1 Institute for Automation and Applied Informatics, Karlsruhe Institute of Technology (KIT), Karlsruhe, Germany; 2 Department of Ophthalmology, Rostock University Medical Center, Rostock, Germany; 3 Augenarztpraxis Spremberg, Carl-Thiem-Klinikum-Poliklinik GmbH (MVZ), Cottbus, Germany

## Abstract

The capability of corneal confocal microscopy (CCM) to acquire high-resolution *in vivo* images of the densely innervated human cornea has gained considerable interest in using this non-invasive technique as an objective diagnostic tool for staging peripheral neuropathies. Morphological alterations of the corneal subbasal nerve plexus (SNP) assessed by CCM have been shown to correlate well with the progression of neuropathic diseases and even predict future-incident neuropathy. Since the field of view of single CCM images is insufficient for reliable characterisation of nerve morphology, several image mosaicking techniques have been developed to facilitate the assessment of the SNP in large-area visualisations. Due to the limited depth of field of confocal microscopy, these approaches are highly sensitive to small deviations of the focus plane from the SNP layer. Our contribution proposes a new automated solution, combining guided eye movements for rapid expansion of the acquired SNP area and axial focus plane oscillations to guarantee complete imaging of the SNP. We present results of a feasibility study using the proposed setup to evaluate different oscillation settings. By comparing different image selection approaches, we show that automatic tissue classification algorithms are essential to create high-quality mosaic images from the acquired 3D datasets.

## Introduction

Diagnosis of peripheral neuropathies currently relies on assessments of nerve function that commonly detect pathologic alterations only after the manifestation of clinical symptoms. In this context, novel objective diagnostic methods with increased sensitivity are urgently needed to facilitate early diagnosis and to evaluate novel therapeutic approaches. Confocal laser scanning microscopy of the cornea is a promising novel approach which allows non-invasive *in vivo* imaging of the corneal subbasal nerve plexus (SNP) and therefore enables direct assessment of its morphology^1–3^. In recent years, corneal confocal microscopy (CCM) has been used to reveal significant morphological changes of the SNP in diverse diseases such as diabetic peripheral neuropathy (DPN), chronic migraine^4^, multiple sclerosis^5,6^ and familial amyloid neuropathy^7^. DPN in particular has been the main focus of various studies^8^. Since characteristic morphological alterations of the SNP already occur at an early stage of DPN^9,10^, CCM has the potential to provide reliable biomarkers for the early assessment of DPN. A major limitation of this imaging technology arises from the inhomogeneous distribution of nerve fibres across the area of the cornea^11^. The field of view of a single CCM image (typically 0.16 mm²) is therefore insufficient for the reliable morphometric characterisation of the SNP. Recommendations in the literature to analyse samples of multiple non-overlapping CCM images to effectively expand the examined corneal area range from three^12^ to eight^13^ images, which have to be manually selected from a larger set of acquired images according to predefined quality criteria.

An alternative approach to increase the examined area is to compose a mosaic image from several overlapping CCM images. Various algorithms for the creation of mosaic images have been proposed^14–20^: Some rely on the operator to acquire appropriate image sequences manually, while others incorporate more automated methods for capturing an extended area in the recorded image sequence. The so-called EyeGuidance system that we described in preceding articles^21,22^ implements a fully automated image acquisition process. A computer-controlled fixation target, presented to the contralateral eye and moving in an outward-spiralling pattern, guides the eye movements of the patient during the entire image acquisition process. Using this approach, an average expansion rate of the acquired mosaic image area of 0.16 mm²/s can be achieved. Based on the examination of the progression of morphological SNP parameters in continuously expanding mosaic images, we recommended a minimum size of the mosaic images of 1.5 mm² for a reliable morphological characterisation of the SNP^23^, which requires a mean examination time of less than 10 seconds^21^ with the EyeGuidance system.

**Figure 1:**
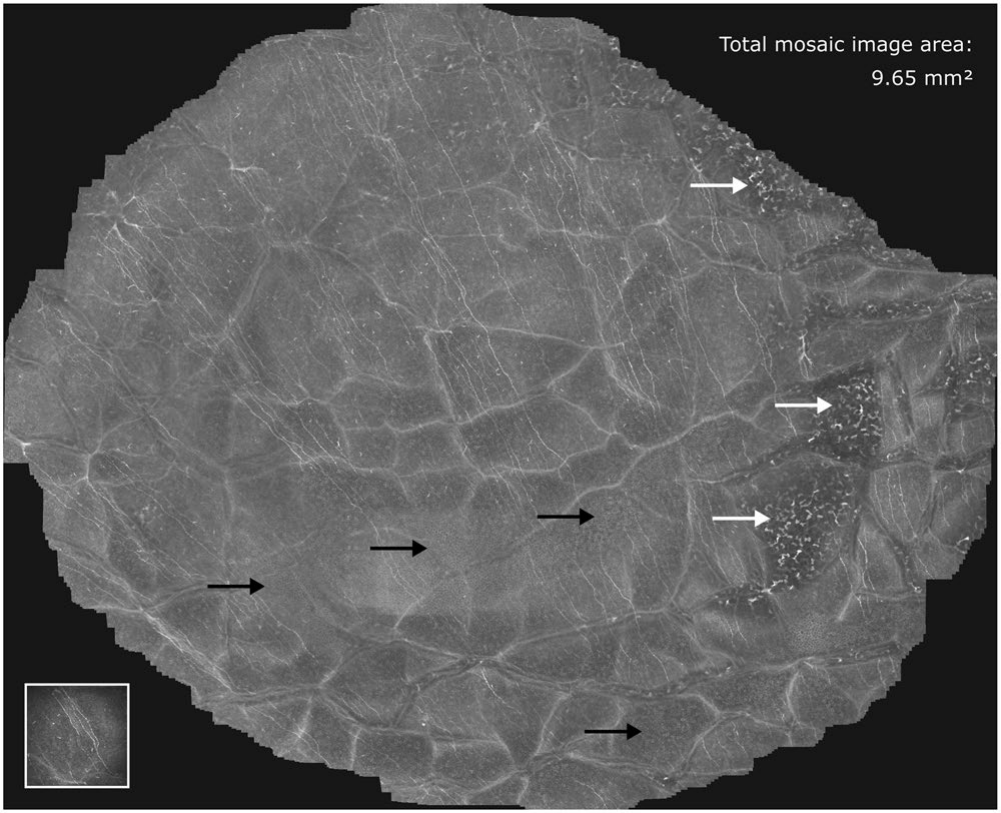
SNP mosaic image created using the EyeGuidance system without focus adjustments, as described by Allgeier *et al*.^21^. Several image areas show epithelium (black arrows) or stroma tissue (white arrows). The bottom left inset represents the size of a single CCM image (0.4 × 0.4 mm²) from the recorded dataset.

Due to the limited depth of field, which is characteristic to confocal microscopy, it cannot be guaranteed that all the recorded CCM images are well focused on the SNP layer. Even slight displacements perpendicular to the focus plane during the image acquisition process or small deviations from the surface-parallel arrangement of the SNP lead to images that partially or entirely contain extraneous tissue (epithelium or stroma)^21^. Consequently, the mosaic image generated from such an image sequence does not show SNP in the complete field of view (see Fig. 1), which ultimately leads to incorrect morphological SNP parameter measurements. This issue is also common to almost all of the previously proposed mosaicking methods^14–18,21^ as they are inherently 2D processes without the ability of (controlled) focus adjustments. Only Lagali *et al*.^19,20^ describe a mosaicking process with manual focus variation during the image acquisition process; images that do not show SNP are sorted out manually afterwards.

In order to overcome the issue described above and to realise a reliable, highly automated SNP mosaicking process, we propose a novel approach for automatic online SNP focus tracking in the present article. To this end, the confocal microscope is equipped with a computer-controlled piezo drive unit that performs an oscillating movement of the focus plane during the image acquisition process. We present results from a feasibility test of the proposed setup in conjunction with the EyeGuidance system^22^. The aim of the present study is to examine the potential benefit in mosaic image quality and to evaluate the main process parameter of the focus oscillation, i.e. the oscillation velocity, and to compare three different approaches to create mosaic images from the recorded image sequences.

## Methods

CCM imaging was performed using a Heidelberg Retina Tomograph II (HRT II, Heidelberg Engineering GmbH, Heidelberg, Germany) in conjunction with a modified Rostock Cornea Module (RCM) and the EyeGuidance system^21,22^ to guide the eye movements of the examined person. The study received appropriate ethics committee approval from the institutional review board (Rostock University Medical Centre Ethics committee) in accordance with the Declaration of Helsinki. All examined volunteers gave their informed consent and – in accordance with the ethics committee approval – did not have any known neuropathic or ocular diseases.

### Modified rostock cornea module

Compared to the original RCM^1^ (Heidelberg Engineering GmbH, Heidelberg, Germany), the modified RCM is additionally equipped with a piezo driven actuator (MIPOS 600 SG DIG, Piezosystem Jena GmbH, Jena, Germany) to move the internal objective lens. Due to the changed local arrangement of the lenses of the RCM objective, the square field of view decreases from 0.160 mm² to 0.123 mm².

**Figure 2:**
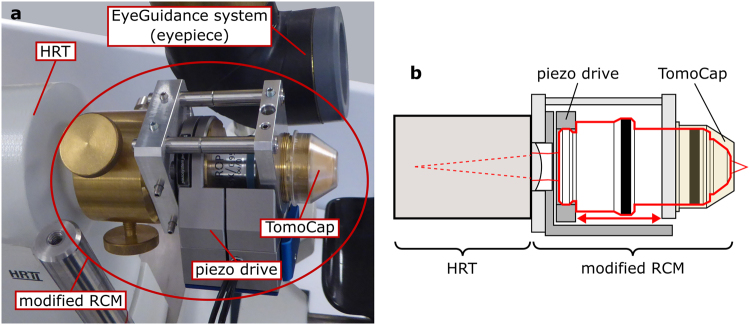
The modified Rostock Cornea Module (RCM). (**a**) Photograph of the modified RCM attached to the HRT II and next to the eyepiece of the EyeGuidance system. (**b**) Schematic drawing of the modified RCM; the piezo drive translates the objective (highlighted by red border) to effect focus changes; the contact TomoCap remains fixed in the process.

The piezo drive has a setting range of 500 µm in closed loop control and can be controlled by a computer via an RS232 interface. An in-house developed adapter device integrates the piezo actuator into the RCM (see Fig. 2); due to the compact design, the piezo drive unit does not interfere with the CCM examinations.

### EyeGuidance

To control the movements of the examined eye during the CCM image acquisition process, the EyeGuidance system presents a computer controlled fixation target to the contralateral eye. A smartphone (Motorola Moto G, Motorola Mobility Germany GmbH, Idstein, Germany) with an Android operating system is used to display the fixation target. The 4.5″ display has a pixel resolution of 1280 × 720. A special optical setup composed of four convex lenses and a tilted mirror realises a compact design suitable for CCM. Three linear axes allow the precise alignment of the eyepiece of the EyeGuidance system. A more detailed technical description of the EyeGuidance hardware setup has been published^22^.

The EyeGuidance smartphone is connected to the controlling PC via USB. The parameters for the fixation target (e.g. path layout, size, colour, speed, acceleration) are defined in an XML file. During the CCM image acquisition process, the EyeGuidance software continuously calculates the position of the fixation target and sends it to the EyeGuidance App running on the smartphone. The update frequency depends on the PC performance; for the setup used in the present study, we measured an update frequency of the fixation target position of approximately 923 Hz, which reliably guarantees a smooth motion of the fixation target even at high velocities.

### CCM setup with modified RCM and EyeGuidance

**Figure 3:**
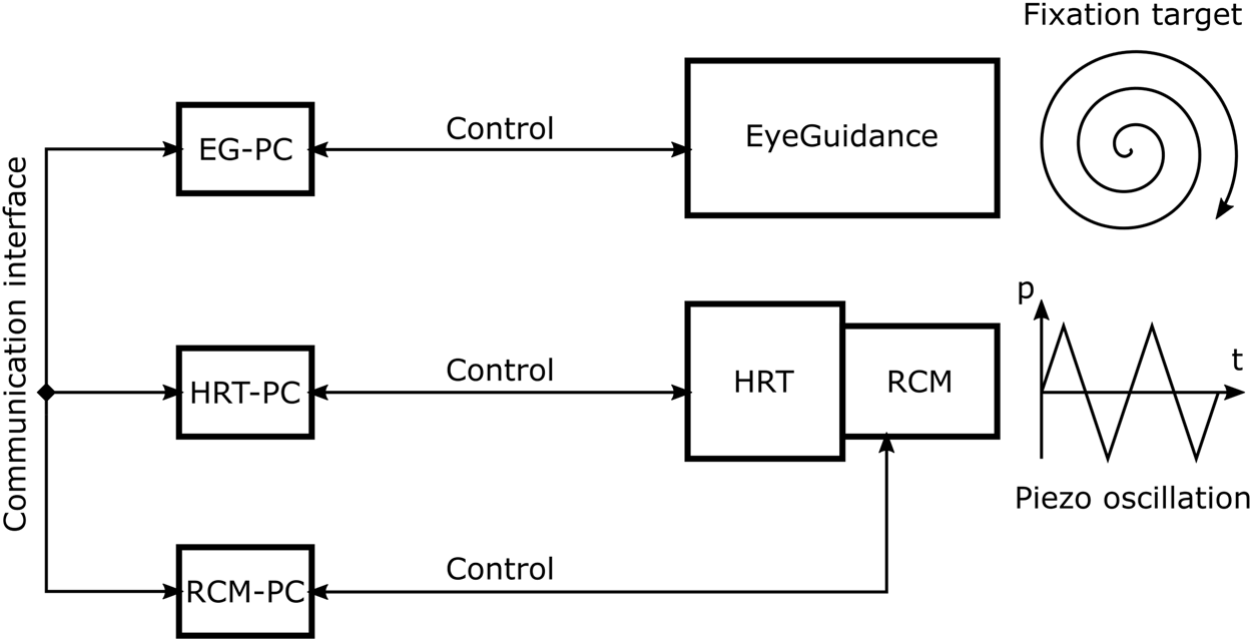
Schematic illustration of the CCM setup with focus plane control and guided eye movements.

The schematic illustration of the CCM setup is shown in Fig. 3. Three dedicated PCs, connected via a TCP/IP network, are used to control the HRT II, the EyeGuidance system, and the modified RCM. The complete CCM image acquisition process is controlled via the HRT computer (HRT-PC, see Fig. 3) as described below.

### CCM image acquisition process

As a preparation step the cornea of the examined eye was locally anaesthetised by instilling Proparakain 0.5% eye drops (Ursapharm, Saarbrücken, Germany) and the Vidisic gel (Bausch & Lomb/Dr. Mann Pharma, Berlin, Germany) is used as a coupling medium between objective and TomoCap as well as between TomoCap and cornea. After positioning the head of the patient in the forehead support, the operator aligns the EyeGuidance system in front of the not examined eye. The operator adjusts the HRT in the conventional way to achieve frontal SNP images of the central cornea. A joystick connected to the piezo drive offers a fine tuning of the focus.

**Figure 4:**
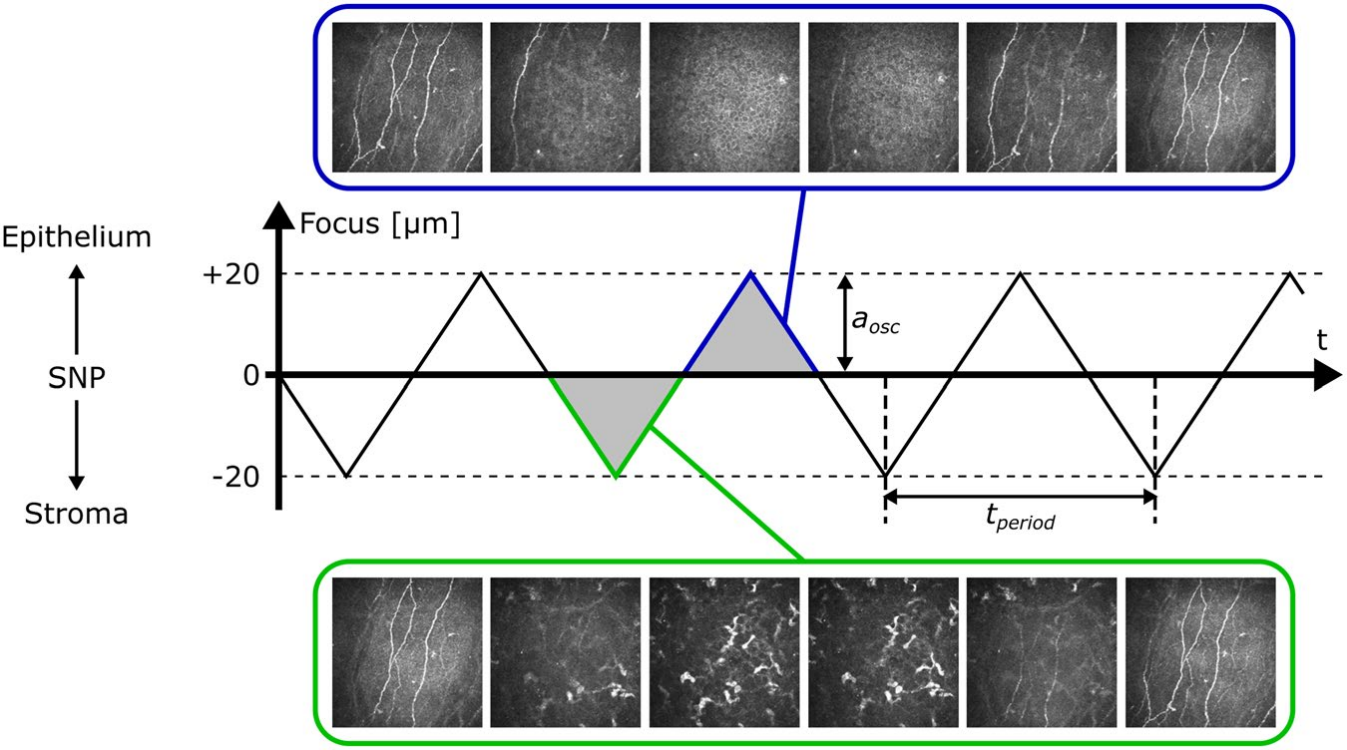
Oscillation of the focus plane during image acquisition. The focus starts in the SNP layer, and then describes a periodic triangular function.

Following these manual initialisation steps, the operator starts the fully automated image acquisition process with the start button, which triggers a signal to EG-PC and RCM-PC. The movement of the EyeGuidance fixation target and the piezo oscillation start simultaneously. The fixation target moves with the predefined speed along an outward spiralling pattern with constantly spaced spiral windings. The piezo drive constantly oscillates with an amplitude *a*_*osc*_ = 20 µm and a constant speed *v*_*osc*_ along the slopes, describing a periodic triangular function (see Fig. 4). The period length is then given as *t*_*period*_ = 4*a*_*osc*_/*v*_*osc*_. The operator terminates the image acquisition process using the stop button after *t*_*exam*_ = 40 seconds, or earlier, if visual assessment of the live image quality is deemed unsatisfying. In that case, the examination is repeated.

In order to assess the influence of the focus oscillation parameters on the acquired image data and the resulting mosaic images, we performed repeated examinations with different settings for *v*_*osc*_ and the velocity of the fixation target *v*_*targ*_. Table 1 lists the absolute values used for the examinations E1 (slow focus oscillation and fixation target movement) through E4 (fast focus oscillation and fixation target movement).

**Table 1:** Process parameters for examinations E1 through E4.

	E1	E2	E3	E4
Focus oscillation velocity *v*_*osc*_ [µm/s]	60.6	121.2	181.8	242.4
Focus oscillation amplitude *a*_*osc*_ [µm]	20	20	20	20
Focus oscillation period *t*_*period*_ [s]	1.32	0.66	0.44	0.33
Fixation target velocity *v*_*targ*_ [pixel/s]	25.5	51	76.5	102

### Mosaicking process

In a post-processing step, mosaic images are calculated from the acquired image sequences. We use in-house developed algorithms to align the single images and to correct motion artefacts^24–26^. In order to create mosaic images from datasets with guided eye movements we developed a special image alignment process suitable for datasets recorded with a fixation target movement on an outward spiralling pattern^21^.

As a first step, all images of a sequence were used for image alignment and correction of motion artefacts. Then, three different approaches for the montage of the mosaic image were implemented and applied to each dataset. The first approach (M1) considers all images of a dataset for the mosaic montage. A second mosaic image (M2) was created using only images from the oscillating focus sequence that had been recorded at a reference focus level. By definition of the process protocol, the imaging process starts in the SNP layer, thus this level was used as reference. Only one image was included from each slope of the oscillation process. In effect, this approach selects a 2D plane from the 3D image data, emulating the EyeGuidance process without focus oscillation^21^ and therefore facilitating a direct comparison of both approaches in terms of mosaic image quality. The third image selection approach (M3) used an automated tissue classification algorithm that had been trained to classify CCM images as SNP or other corneal tissue^27^. Only images classified as SNP were incorporated into the M3 mosaic images.

### Data Availability

The datasets generated during the current study are available from the corresponding author on reasonable request.

## Results

### Data Acquisition

The present study included nine volunteers that were recruited from the clinic staff. All volunteers underwent the four examinations described in the Methods section. The imaging duration was 40 seconds for all examinations. Because of a very limited time slot of one volunteer, we decided to reduce the imaging duration to 30 seconds for all examinations for that person. According to our examination protocol, an examination was immediately repeated if the quality of the acquired image dataset was deemed insufficient based on the visual assessment of the live image during image acquisition. This was not the case for E1, E2 or E3, but for two volunteers in E4. In one of these two cases, we used the second of the two acquired datasets. In the other case, we stopped examination E4 after three unsuccessful attempts. Of the remaining eight successfully acquired datasets from E4 examinations, we had to exclude an additional one since it proved to be unsuitable for automated mosaic image generation due to insufficient image quality that originated from inhomogeneity in the contact gel, which was not recognised during image acquisition. The following results are therefore based on nine datasets for examinations E1 through E3 and the seven datasets from examination E4 that are suitable for mosaic image generation.

### Mosaic Images

Three mosaic images M1, M2 and M3 were created from each of the datasets. Including all images, process M1 of course generates the maximum mosaic image size for all examinations (see Table 2). The number of images used for M2 is defined by the number of oscillation periods, *t*_*exam*_/*t*_*period*_, and therefore increases from 72.0 (5.7% of the recorded images) for E1 nearly proportionally to 240.1 (20.3% of the recorded images) for E4. Compared to M1, the average size of the M2 mosaic images is reduced about 16.6%, 11.0%, 8.1% and 6.4% for examinations E1, E2, E3 and E4, respectively. For M3, the percentage of images used is similar for all examinations (between 36.3% and 39.7%) and the ratio of the average image size between M3 and M1 lies in the range of 91% to 95%.

**Table 2:** Mosaic image sizes and area expansion rates for examinations E1 through E4.

		E1	E2	E3	E4
M1	Average mosaic image size [mm²]	1.34	2.44	3.39	4.62
	Average expansion rate [mm²/s]	0.033	0.062	0.089	0.117
	Average number of images used	1245.8	1187.3	1119.9	1178.7
M2	Average mosaic image size [mm²]	1.12	2.18	3.12	4.31
	Average expansion rate [mm²/s]	0.028	0.055	0.082	0.109
	Average number of images used	72.0	127.1	175.2	240.1
M3	Average mosaic image size [mm²]	1.25	2.30	3.09	4.38
	Average expansion rate [mm²/s]	0.031	0.059	0.081	0.111
	Average number of images used	482.4	469.2	406.3	468.7

**Figure 5:**
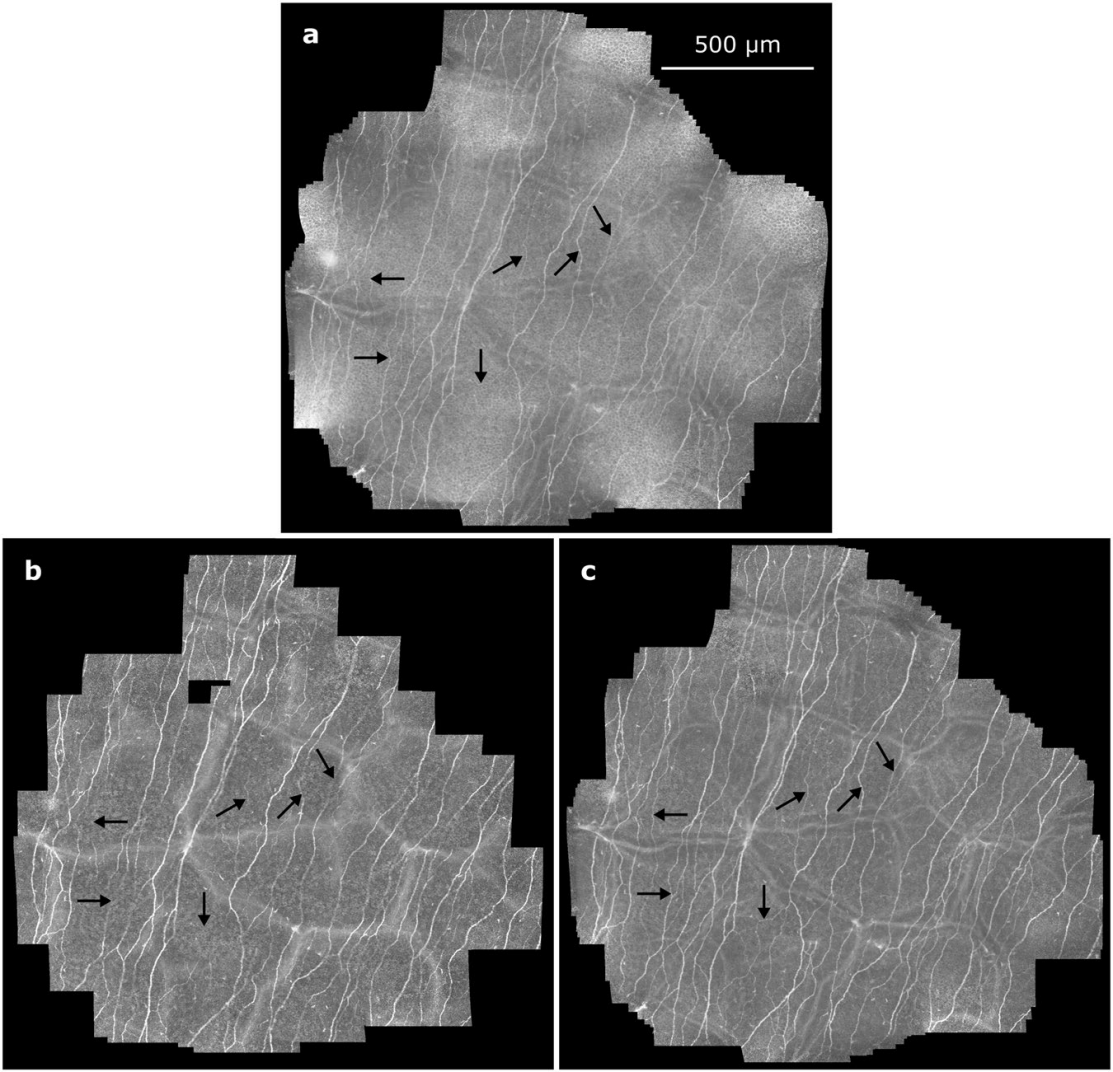
Mosaic images generated from a dataset (subject 8, examination E2); (**a**) mosaic image M1 using all 1245 images; (**b**) mosaic image M2 using the subset of 130 images acquired on the initial focus level; (**c**) mosaic image M3 using the subset of 610 images classified as SNP tissue. The arrows denote nerves that are visible in mosaic image M3 but not in M2.

**Figure 6:**
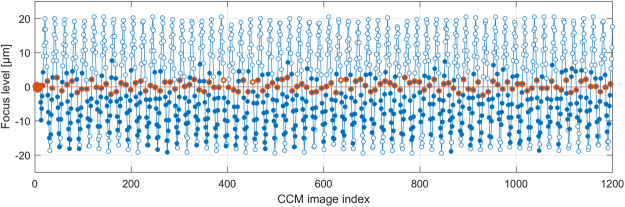
Plot of the focus levels of the CCM images (circles) of the dataset shown in Fig. 5. Red circles mark images acquired on the initial focus level. Solid circles mark CCM images classified as containing SNP tissue. These tend to lie below the initial focus level, and consequentially the M2 mosaic image in Fig. 5b tends towards showing epithelium tissue.

Figure 5 depicts representative examples for the mosaic images created from a single dataset with the processes M1, M2 and M3 (high-resolution versions of these images as well three additional examples from other datasets are available as Supplementary Figures S1 through S4). The mosaic images were evaluated qualitatively with regard to the visibility of the nerve structures of the SNP. Due to the oscillating movement of the focus plane, mosaic images created from all images in a dataset (M1, see Fig. 5a) always show a superimposition of epithelial tissue, nerve structures in the SNP layer, structures from Bowman’s layer (so-called K-structures) and the keratocyte nuclei of the stroma. Most nerve structures are visible but provide only low contrast because of the superimposition of several tissues at most image locations. Where SNP nerves are visible in mosaic images created from the subset of images acquired on the reference focus level defined by the start of the imaging process (M2, see Fig. 5b and red circles in Fig. 6), they commonly possess very good contrast. However, the proportion of the mosaic image area that is optimally focused on the SNP layer varies widely. Some M2 mosaic images are focused well over almost the entire image field; others contain large regions of non-basal epithelial or stroma tissue that encompass more image area than SNP regions. The mosaic images created from the subset of images classified as SNP (M3, see Fig. 5c and solid circles in Fig. 6) show at least the nerves structures that are discernible in the M1 mosaic image. In contrast to the M1 montaging process, the majority of images containing non-SNP tissue is excluded prior to montaging the M3 mosaic images; according to the numbers of images used for the respective mosaicking processes (see Table 2), the proportion of excluded images ranges between 60.3% and 63.7% of all images. Consequently, the nerve structures manifest in M3 with good contrast against the surrounding tissue. The exclusion of images leads to the above-mentioned reduction of the field of view of the M2 and M3 mosaics.

## Discussion

**Figure 7:**
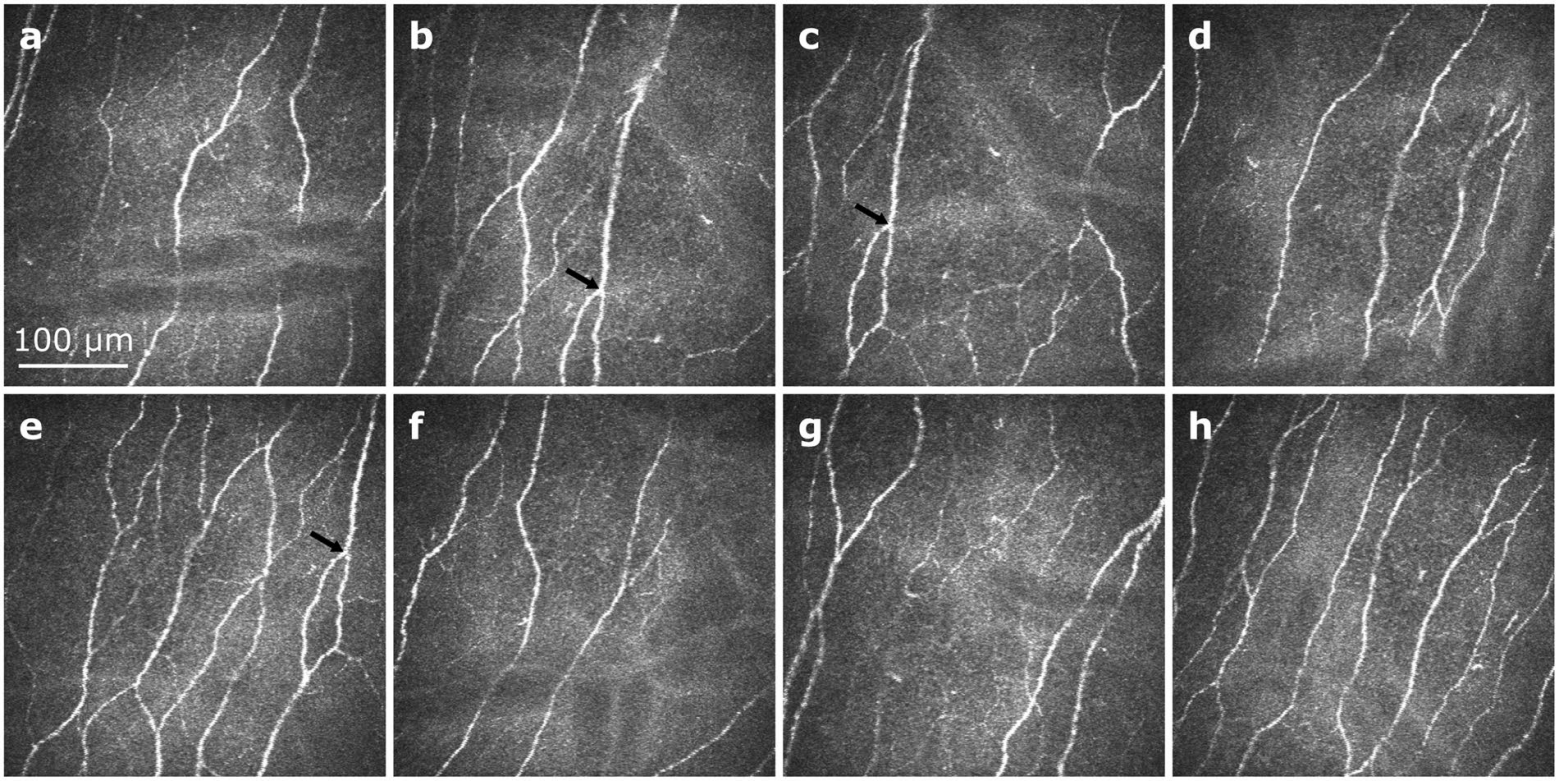
Exemplary sample of eight CCM images of the SNP manually selected from the dataset used for Fig. 5 (subject 8, examination E2, 1246 images total). Repeated presence of the same SNP region in multiple images (as for example in subfigures (**b**,**c**,**e**) see black arrows) can potentially introduce bias effects to morphometric parameter measurements and should therefore be avoided; identification of such repeatedly represented SNP regions in a manual image selection procedure is not trivial.

Although the majority of CCM studies of the SNP published in literature are based on the examination of sets of single CCM image frames, the number of publications on wide-field CCM image creation by different research groups has increased in recent years. Both the CCM-frame approach and the wide-field approach have their unique properties^12,19^. The most important advantages of the CCM-frame approach are the relatively straightforward and well-established image acquisition process and the good support for morphometric analysis of the standard-sized and -shaped CCM frames^12^ by established software. Its major disadvantage is the necessity for manual selection of non-overlapping CCM frames from a larger pool of up to several hundreds of CCM images^12,28^ that are recorded during the image acquisition phase; see Fig. 7 for a sample of eight SNP images manually selected from the same dataset used for Fig. 5. This step is time-consuming, requires expert knowledge and is prone to subjective bias effects^12,19^. By contrast, the wide-field approach is commonly more complex in terms of the software^12,14–21^ and hardware^17,21^ setup. Semi-automatic morphometric analysis of large mosaic images has been described as more laborious compared to standard CCM frames^12^, although further developments towards fully-automatic software tools can provide a solution^19,20^. A major advantage of the wide-field approach, on the other hand, is its capability to provide image data within a larger field of view, potentially allowing the clinician to repeatedly assess identical tissue regions^19^. Regarding the diagnostic value of both general approaches, Kheirkhah *et al*. find no significant differences^12^, whereas Lagali *et al*. point out potential benefits in morphometric analysis of wide-field mosaic images^19^.

Our aim is to develop a reliable, easy-to-use and fast tool for imaging and visualising an expanded area of the SNP. The 2D-only EyeGuidance process (2D-EG) without focus adjustment, originally published in 2014^21^, achieved an average area expansion rate of approximately 0.16 mm²/s. Those examinations also established that a high velocity of the fixation target movement does not negatively influence the resulting mosaic image quality but has positive effects regarding the patient compliance, by decreasing the required examination time and because fast target movement is perceived as more comfortable than slow movement. Although already yielding promising results, the primary limitation of the 2D-EG process is its inability to compensate deviations of the focus plane from the SNP layer, which are encountered in practice for multiple reasons and are difficult or even impossible to avoid^21^.

The focus of the present study lies on the evaluation of an automated oscillation of the focus plane during image acquisition, effectively extending the process into the third dimension (3D-EG). The main process parameter that changes between the examinations E1 through E4 is the focus oscillation velocity *v*_*osc*_. The fixation target velocity was adjusted such that the fixation target translation throughout an oscillation period always remained constant, i.e. the ratio between *v*_*osc*_ and fixation target velocity was identical for all examinations (see Table 1). The relation between the two velocities was chosen such that after one focus oscillation period (corresponding to two passes through the SNP layer) the resulting lateral displacement between the CCM images was approximately 200 µm.

The first objective was to analyse the examination duration, as this is essential for patient comfort and compliance. As expected, the measured average area expansion rates (see Table 2) are directly proportional to the speed of the fixation target respectively the oscillation velocity *v*_*osc*_ (see Table 1). An average area expansion rate of 0.11 to 0.12 mm²/s (depending on the mosaicking approach used) was measured for the fastest oscillation velocity, which is a decrease of only 26–31% compared to the 0.16 mm²/s of the 2D-EG process^21^. This is an interesting and unexpected finding, considering that the field of view of the modified RCM is reduced about 23% and the new 3D-EG process essentially covers a volume of tissue extending 40 µm in depth, instead of just a single plane. Regarding the question, whether *v*_*osc*_ can be increased further in order to accelerate the imaging process, it is important to look at the translation of the focus plane between two adjacent images. In examination E4, the focus plane moves about 8 µm during the recording of a single image. This distance is already in the order of the depth of field of the microscope^29^. Increasing the *v*_*osc*_ beyond the value used for E4, although technically possible, is not practicable because it would no longer be guaranteed that at least a single image of the SNP layer is acquired along each slope of the focus oscillation process. Further acceleration of the imaging process can therefore only be achieved by decreasing the oscillation amplitude.

Regarding the reliability of the imaging process, we did not find any qualitative difference between examinations E1 through E3. All of those examinations were successfully completed at the first attempt. In contrast, examination E4 had to be repeated for two volunteers due to unsatisfactory quality of the dataset, and we finally only obtained seven mosaic images from the nine volunteers, as described in the Results section. For one of the two missing datasets, the exclusion could be attributed to inhomogeneity in the contact gel, which happens during preparation of the device. This exclusion is therefore unrelated to the process parameters of the oscillation process. The noticeable increase in the number of repetitions in E4 might be related to the process parameters, i.e. the higher oscillation and fixation target velocities. However, as the order of the examinations was not randomised and the other three examinations always preceded E4, it may also be an effect of diminishing participant compliance over time. Finally, considering the relatively low number of participants, no correlation can be established between *v*_*osc*_ and the reliability of the imaging process based on the analysed data.

Based on the proportional relationship between oscillation velocity and average area expansion rate stated above, we conclude that faster oscillation velocities possess the advantage of reducing the required examination time and allow faster fixation target movement, which is perceived as more comfortable by the examined volunteers. Further investigation is required to find whether a very high *v*_*osc*_ in the order of the velocity used for E4 reduces the reliability of the imaging process. However and in any case, even the process parameters of examination E3 allow an average expansion rate of covered SNP area of more than 0.08 mm²/s and facilitate an examination duration of less than 20 seconds for a target area of 1.5 mm².

The second objective of the present study was to assess the mosaic image quality. No systematic differences were found between examinations E1 through E4. The differences in mosaic image quality between the mosaicking approaches M1 through M3, however, are obvious (see Fig. 5 and Supplementary Figures S1 through S4). The comparative qualitative evaluation of the three presented mosaic montaging processes has to be guided by the question of how the characteristics of the resulting mosaic images affect the reliable quantitative morphometric characterisation of the SNP. Two potential detrimental factors play a major role in this regard:

- incorrect nerve fibre segmentation, encompassing both unsegmented nerve structures (false negatives) and segmented structures that are not nerve fibres (false positives) and
- incorrect tissue regions, i.e. mosaic image regions showing tissue from above or below the SNP layer.

The second point is of particular interest as, even assuming a perfect segmentation procedure, such incorrect tissue regions lead to an underestimation bias of the morphometric parameter measurements, which are commonly normalised to the assessed area (cf. corneal nerve fibre length, corneal nerve fibre density, corneal nerve branch density^13,24,30,31^). Incorrect tissue regions represent image area without adding any nerve fibres or branches.

These two points clearly reveal the deficits of the mosaicking approaches M1 and M2. The M1 mosaics contain practically no regions with no SNP tissue visible, which of course is the intent of the focus oscillation during the imaging process. However, the low contrast of the nerve structures in the mosaics is counterproductive for correct nerve fibre segmentation and therefore carries the risk for increased deviations of the measured SNP parameters compared to the M2 and M3 mosaics. In some cases, the contrast can even be reduced to a level where the nerve fibres almost disappear, although nerves have been imaged at the respective location, as evidenced by the corresponding M3 mosaic (see Fig. 5).

As the M2 mosaics are composed from images from a specific, constant focus depth, they manifest a very similar characteristic as the ones that result from the originally proposed 2D-EG process^21^. Their good contrast supports the correct segmentation of nerve structures. Contrary to the other two mosaicking approaches, however, they can potentially contain ill-focused image regions and consequentially carry the risk of reducing the accuracy of the measured SNP parameters.

The M3 mosaics combine the advantages of both of the other approaches while at the same time avoiding the disadvantages: They provide satisfactory contrast of the nerve fibres in order to support reliable nerve segmentation and they do not contain ill-focused tissue regions. Such regions can only be avoided by continually controlling the focus, which cannot be adjusted manually while at the same time minimising the duration of examination^21^. The advantageous effects of small focus variations on the wide-field visualisation of the SNP are in general accordance with the conclusions drawn by Lagali *et al*.^19^.

The primary purpose of the present article is to provide a detailed description of the highly automated 3D-EG process and to evaluate its technical parameters. The results demonstrate the feasibility of the process and the qualitative advantages with regard to SNP visualisation, as compared to 2D-only mosaicking approaches. Further evaluation of the diagnostic utility of the 3D-EG process was not within the scope of the presented feasibility study, therefore no morphometric parameters were calculated in the mosaic images. However, two previously published results from larger studies have to be taken into account: the reported absence of significant quantitative differences between SNP morphometry measurements in sets of single CCM frames and in wide-field images^12^, and the reported benefits of systematic focus variations on the resulting wide-field images of the SNP^19^. Given these previous results, the diagnostic utility of the presented 3D-EG process can be assumed to be at least equal to other published CCM techniques.

A further limitation of the presented system is the complex setup with three connected PCs (see Fig. 3), which is not suitable in clinical practice. This setup originates from the distributed development of the software modules. For a potential future system intended to be used for wide-field CCM in a clinical environment, it is necessary to integrate all software modules into a single, dedicated PC system.

In the current implementation of the described process, the mosaicking step can only start after the entire image dataset has been recorded. The quality of the imaging process has to be estimated based on the rapidly changing live image (see Supplementary Videos S5 through S8), which requires a lot of expert knowledge and is prone to misjudgement. With a potential real-time mosaic image visualisation, the operator could directly assess the quality of the growing mosaic image. This could allow earlier termination and repetition of the examination and reduce overall examination time for the patient. Future development work will therefore be dedicated towards realising an online mosaicking process with tissue classification^27,32^ and a real-time control of the focus oscillation parameters. The purpose of this control scheme is to reduce the oscillation amplitude and continuously adjust the zero-position of the oscillation in order to reduce the examination duration further while at the same time reliably keeping the SNP layer inside the oscillation range (see Supplementary Video S9 for a simulated animation of online mosaicking with tissue classification).

As the presented process captures image data extending into the third dimension, reconstructing a volume representation of the acquired tissue region could provide valuable additional information, not only for imaging the SNP but also potentially for other tissues. This will also be an interesting topic for future research work.

In conclusion: We proposed a novel concept for automated wide-field CCM imaging of the SNP and provided results of the first feasibility study. Using an externally mounted, piezo-driven focus control unit, we realised a fast 3D imaging process that facilitates the reliable acquisition of the SNP and adjacent tissue layers. A tissue classification algorithm filters the CCM datasets, keeping only images of the SNP layer. The direct comparison of mosaic images composed of these SNP-filtered 3D datasets against mosaic images montaged from a 2D plane clearly reveals the advantages of the proposed concept over 2D-only mosaicking approaches.

## Electronic supplementary material

Supplementary Figures S1-S4

Supplementary Video S5

Supplementary Video S6

Supplementary Video S7

Supplementary Video S8

Supplementary Video S9
