# Supplementary material for: 3D confocal laser-scanning microscopy for large-area imaging of the corneal subbasal nerve plexus
Source: Sci Rep. 2018 May 10;8:7468. doi: 10.1038/s41598-018-25915-6 (PMC5945773; doi:10.1038/s41598-018-25915-6)
Supplement: Supplementary file 1 — Supplementary Figures S1-S4 [file 41598_2018_25915_MOESM1_ESM.pdf]

3D confocal laser-scanning microscopy for large-area imaging of the corneal subbasal nerve plexus

Stephan Allgeier[1*], Andreas Bartschat[1], Sebastian Bohn[2], Sabine Peschel[3], Klaus-Martin Reichert[1], Karsten Sperlich[2], Marcus Walckling[2], Veit Hagenmeyer[1], Ralf Mikut[1], Oliver Stachs[2], Bernd Köhler[1]

[1]Institute for Automation and Applied Informatics, Karlsruhe Institute of Technology (KIT), Karlsruhe, Germany
[2]Department of Ophthalmology, Rostock University Medical Center, Rostock, Germany
[3]Augenarztpraxis Spremberg, Carl-Thiem-Klinikum-Poliklinik GmbH (MVZ), Cottbus, Germany

*Corresponding author:

Stephan Allgeier
Karlsruhe Institute of Technology (KIT)
Institute for Automation and Applied Informatics
Hermann-von-Helmholtz-Platz 1
D-76344 Eggenstein-Leopoldshafen
Germany

Phone: +49-721-608-23172

e-mail: stephan.allgeier@kit.edu

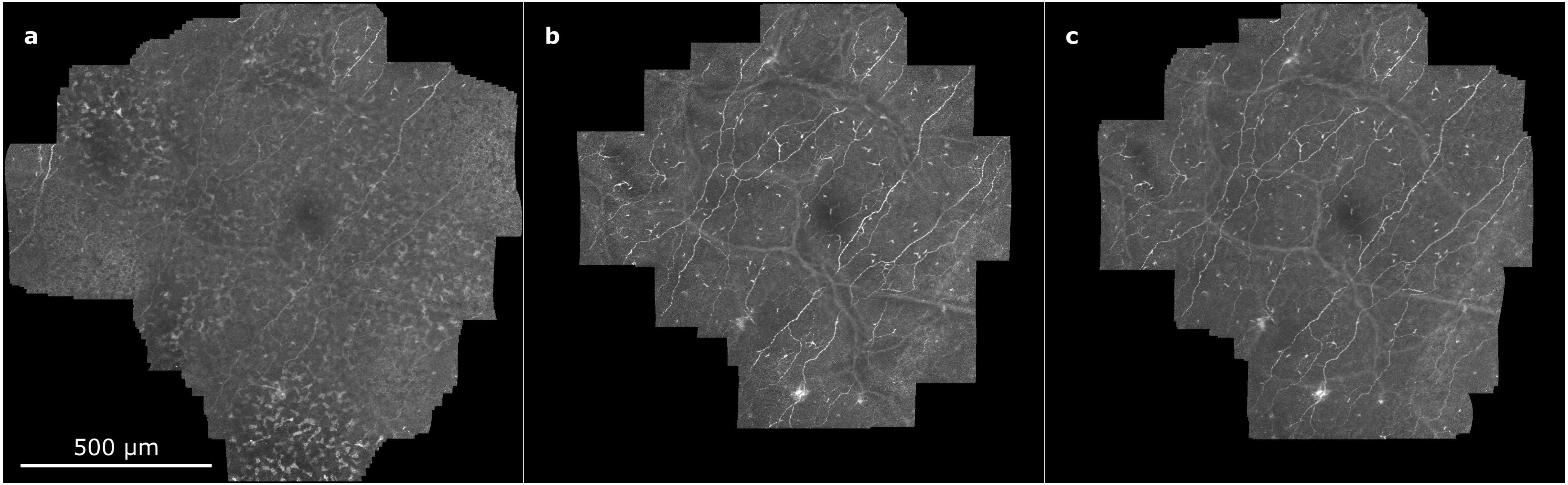

Figure S1: Mosaic images generated from a dataset (subject 1, examination E1); (a) mosaic image M1 using all images; (b) mosaic image M2 using the subset of images acquired on the initial focus level; (c) mosaic image M3 using the subset of images classified as SNP tissue.

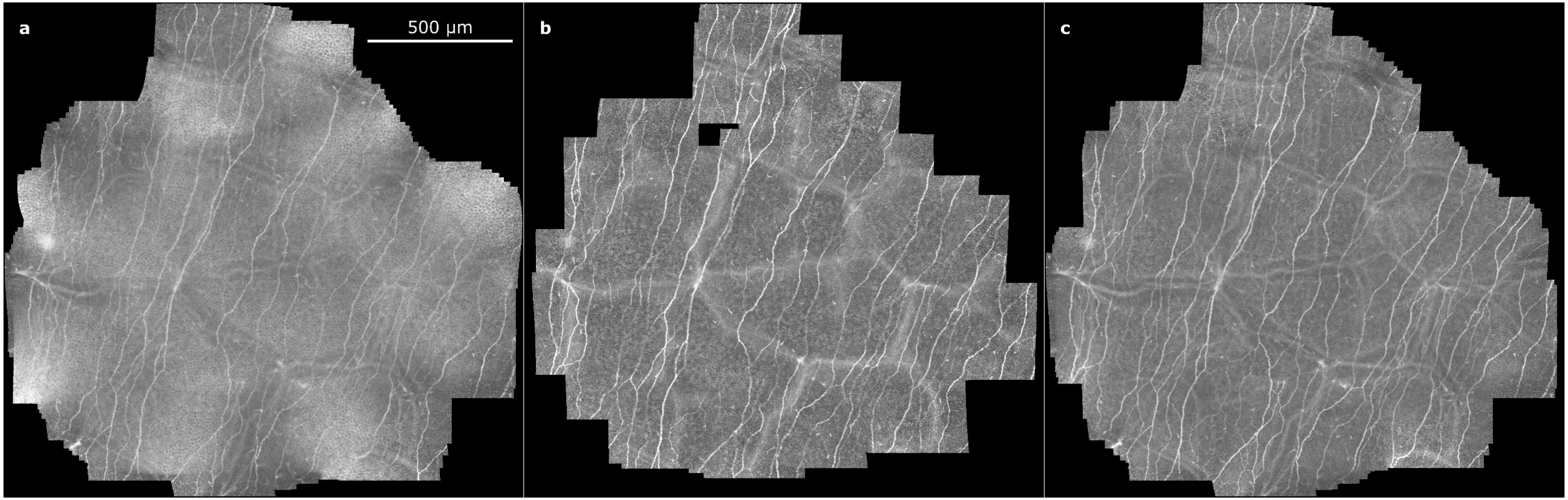

Figure S2: Mosaic images generated from a dataset (subject 8, examination E2); (a) mosaic image M1 using all images; (b) mosaic image M2 using the subset of images acquired on the initial focus level; (c) mosaic image M3 using the subset of images classified as SNP tissue.

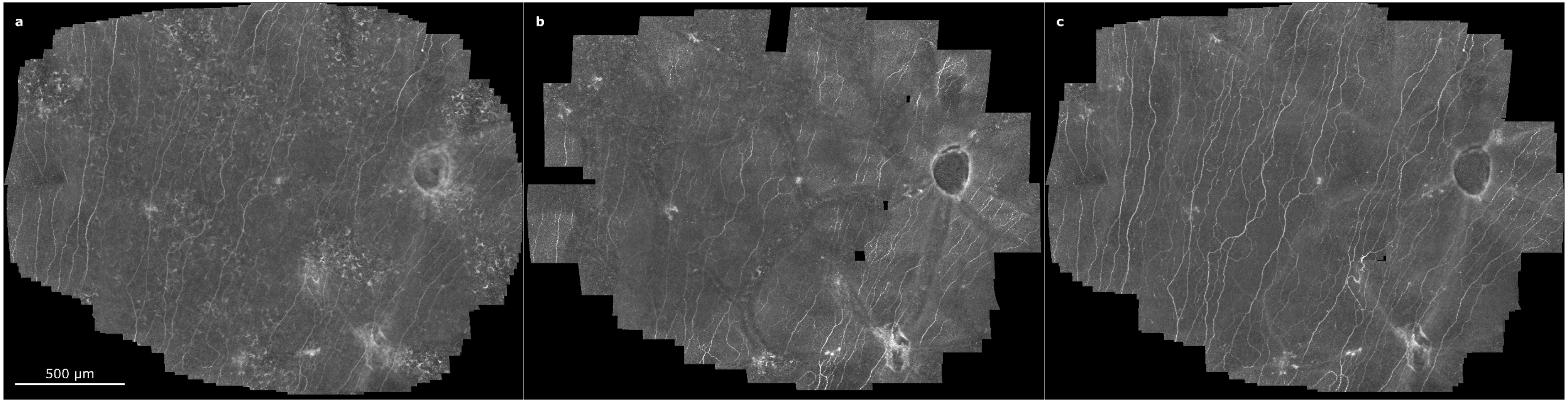

Figure S3: Mosaic images generated from a dataset (subject 6, examination E3); (a) mosaic image M1 using all images; (b) mosaic image M2 using the subset of images acquired on the initial focus level; (c) mosaic image M3 using the subset of images classified as SNP tissue.

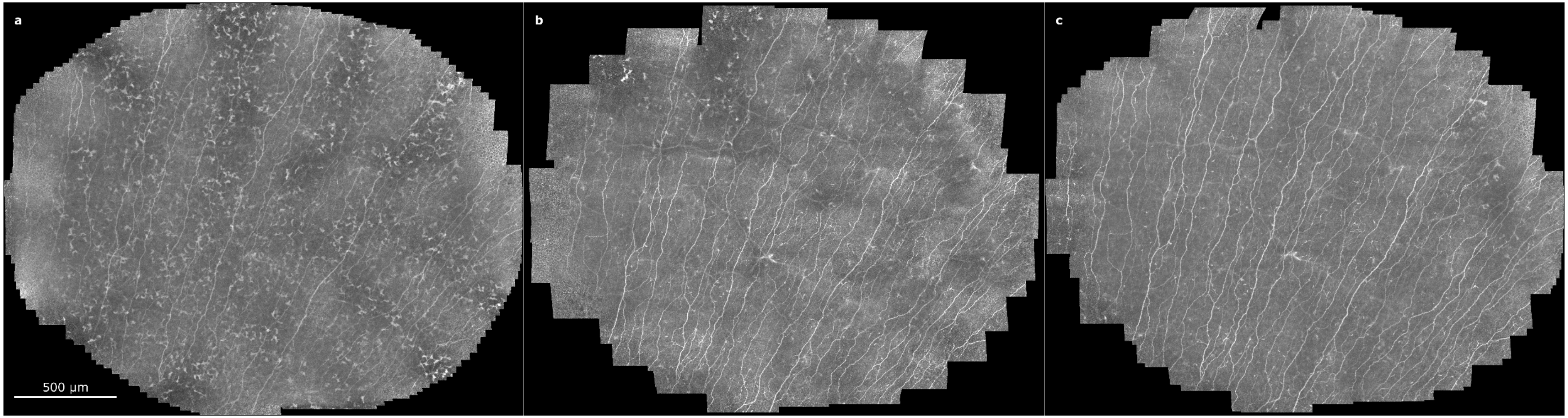

Figure S4: Mosaic images generated from a dataset (subject 9, examination E4); (a) mosaic image M1 using all images; (b) mosaic image M2 using the subset of images acquired on the initial focus level; (c) mosaic image M3 using the subset of images classified as SNP tissue.
